# Probing the enzyme kinetics, allosteric modulation and activation of α1- and α2-subunit-containing AMP-activated protein kinase (AMPK) heterotrimeric complexes by pharmacological and physiological activators

**DOI:** 10.1042/BJ20151051

**Published:** 2016-02-24

**Authors:** Francis Rajamohan, Allan R. Reyes, Richard K. Frisbie, Lise R. Hoth, Parag Sahasrabudhe, Rachelle Magyar, James A. Landro, Jane M. Withka, Nicole L. Caspers, Matthew F. Calabrese, Jessica Ward, Ravi G. Kurumbail

**Affiliations:** * Department of World Wide Medicinal Chemistry, Pfizer Global Research and Development, Eastern Point Road, Groton, CT 06340, U.S.A.; † CVMED Research unit, Pfizer Global Research and Development, 620 Memorial Drive, Cambridge, MA 02139, U.S.A.; ‡ The Department of Pharmacokinetics Dynamics and Metabolism, Pfizer Global Research and Development, Eastern Point Road, Groton, CT 06340, U.S.A.

**Keywords:** allosteric activation, AMP-activated protein kinase (AMPK), heterotrimeric complex, kinase activity, metabolic sensor, recombinant protein

## Abstract

AMP-activated protein kinase (AMPK) is a serine/threonine protein kinase that serves as a pleotropic regulator of whole body energy homoeostasis. AMPK exists as a heterotrimeric complex, composed of a catalytic subunit (α) and two regulatory subunits (β and γ), each present as multiple isoforms. In the present study, we compared the enzyme kinetics and allosteric modulation of six recombinant AMPK isoforms, α1β1γ1, α1β2γ1, α1β2γ3, α2β1γ1, α2β2γ1 and α2β2γ3 using known activators, A769662 and AMP. The α1-containing complexes exhibited higher specific activities and lower *K*_m_ values for a widely used peptide substrate (SAMS) compared with α2-complexes. Surface plasmon resonance (SPR)-based direct binding measurements revealed biphasic binding modes with two distinct equilibrium binding constants for AMP, ADP and ATP across all isoforms tested. The α2-complexes were ∼25-fold more sensitive than α1-complexes to dephosphorylation of a critical threonine on their activation loop (pThr^172/174^). However, α2-complexes were more readily activated by AMP than α1-complexes. Compared with β1-containing heterotrimers, β2-containing AMPK isoforms are less sensitive to activation by A769662, a synthetic activator. These data demonstrate that ligand induced activation of AMPK isoforms may vary significantly based on their AMPK subunit composition. Our studies provide insights for the design of isoform-selective AMPK activators for the treatment of metabolic diseases.

## Abstract (precis)

We have studied enzyme kinetics, nucleotide binding and allosteric modulation of six recombinant AMP-activated protein kinase (AMPK) isoforms by known allosteric activators. α1-Complexes exhibited higher specific activities and lower *K*_m_ values for a peptide substrate, but α2-complexes were more readily activated by AMP.

## INTRODUCTION

AMP-activated protein kinase (AMPK) serves as a metabolic fuel gauge, sensing the energy status of the cell and whole organism [1]. Under conditions of limiting energy reserves or related cellular stresses, AMPK gets activated which leads to inhibition of biosynthetic pathways that consume ATP, and activation of catabolic pathways that enhance ATP production [2,3]. Structurally, mammalian AMPK is a heterotrimeric complex, composed of a single catalytic (α) subunit and two regulatory (β and γ) subunits [4,5]. Multiple isoforms exist for each subunit (α1, α2, β1, β2, γ1, γ2, γ3). The α-subunit consists of an N-terminal catalytic domain, followed by an auto-inhibitory region and a C-terminal β- and γ-subunit interacting domain [6]. The β-subunits contain an N-terminal glycogen/carbohydrate binding domain [7] and a C-terminal region that tethers the β-subunit to the α- and γ-subunits [8] with Gly^2^ of the β1-subunit being modified by co-translational myristoylation [9]. The γ-subunit contains a variable N-terminal region followed by four conserved cystathionine-β-synthase sequence motifs which form Bateman domains 1 and 2 [10]. The Bateman domains constitute the four putative-binding sites for ATP, ADP and AMP [7,11].

Phosphorylation of a critical threonine (Thr^172/174^) on its activation loop (Thr^174^ for α1 and Thr^172^ for α2) within the kinase domain of the α-subunit either by calcium/calmodulin-dependent protein kinase β (CaMKKβ) or by liver kinase B (LKB1), leads to a significant increase (>500-fold higher) in the kinase activity of AMPK [12–14]. Once phosphorylated, the catalytic activity can be further increased allosterically either by AMP [15] or by synthetic small molecule activators such as A769662 [16]. In addition, binding of AMP or A769662 protects the activation loop Thr^172/174^ from dephosphorylation by an inactivating phosphatase [15,17], thereby extending the ‘half-life’ of the active AMPK complex. However, a more recent study has indicated that the allosteric activator A769662 activates the α1β1γ1 complex independent of α-Thr^174^ phosphorylation under conditions where Ser^108^ of the β-subunit is phosphorylated [18]. AMP does not influence phosphorylation of recombinant, non-myristoyled AMPK by upstream kinases [13]. Recent work, however, has also shown that β-myristoylation plays a gatekeeper role in signal initiation by allowing upstream kinases to fully phosphorylate and activate AMPK in response to increases in cellular AMP concentration which is absent from recombinant AMPK expressed in *Escherichia coli* [19].

Previous structural studies with truncated mammalian AMPK regulatory core revealed the presence of four potential nt-binding sites on the γ-subunit, three of which were occupied by AMP (sites 1, 3 and 4) whereas site 2 was empty [20,21]. Of these, site 4 appears to be a structural site that retains a molecule of AMP throughout biochemical purification and is critical for the proper folding of the γ-subunit and the heterotrimeric complex. Although ATP can occupy site 4 *in vitro*, it preferentially binds AMP which appears to be its physiological ligand [21]. Competitive binding studies using fluorescent ATP derivatives have shown that all three nts (AMP, ADP and ATP) can bind to sites 1 and 3 with similar affinities. The binding affinities for the stronger site appear to be 30- to 40-fold higher than the weaker site for all the three nts [22]. Moreover, ATP that is chelated with Mg^2+^ appears to bind ∼10-fold weaker compared with free ATP [22]. Mechanistically, binding of AMP to the γ-subunit allows a small regulatory segment of the α-subunit (α2 residues 365–371) called the α-hook or α-RIM2 to directly interact with bound AMP and create an allosteric conformational change at the catalytic active site. As a consequence, the α-pThr^172/174^ (phosphorylated Thr^172/174^) could be protected from dephosphorylation by phosphatases and sustain its kinase activity for an extended period.

Recently, crystal structures of near full-length AMPK isoforms (α1β2γ1, α1β1γ1 and α2β1γ1) have been published in the presence or absence of synthetic activators [23–25]. These structures have revealed the presence of a novel allosteric site at the interface of the kinase domain of the α-subunit and the carbohydrate binding module (CBM also known as glycogen binding domain or GBD) of the β-subunit. This allosteric pocket which potentially could be a regulatory site for as yet unknown endogenous ligand harbours a binding site for small molecule synthetic ligands, A769662 and compound 991 [25]. Previous hydrogen deuterium exchange (HDX) mass spectrometric studies have shown that binding of A769662 to AMPK causes significant conformational changes in and around the activation loop of the kinase module in the α-subunit [26]. Also, kinetic studies showed that A769662 primarily exerts its effects by lowering the *K*_m_ for the peptide substrate (SAMS) [24]. Crystal structures have revealed the existence of an α-helix at the C-terminal portion of the CBM module which forms a three helical bundle with the B- and C-α-helices of the protein kinase module from the α-subunit. These studies have suggested a molecular mode of action for A769662 and compound 991 whereby the ligands further improve the substrate-binding site through their influences on C-helix of the α**-**subunit kinase domain and through other conformational changes in and around the activation loop. As a consequence, the pThr^172/174^ residue gets sequestered from phosphatases. Thus AMP and A769662 can activate AMPK complexes by distinct molecular mechanisms through binding at opposite poles of AMPK heterotrimeric complex [24].

The existence of multiple isoforms of α-, β- and γ-subunits encoded by seven different genes leads to the theoretical possibility of 12 possible AMPK complexes. Much work has gone into establishing the tissue distribution of the various AMPK isoforms in different species based on mRNA and protein levels. Using an activity-based protein profiling method in tandem with MS, Wu et al. showed that human liver contains both α1β1γ1 and α1β2γ1 isoforms whereas the primary isoform in the rat liver is α2β1γ1 [27]. It has been well established that the predominant β-subunit in human skeletal muscle is β2 which associates with other subunits to generate α2β2γ1 (60%), α1β2γ1 (15%) and α2β2γ3 (20%) complexes in muscle [28]. Moreover, the expression of the γ3-subunit appears to be restricted to skeletal muscle where it is primarily expressed in fast twitch glycolytic extensor digitorum longus (EDL) muscle fibers [28]. It has also been found that a specific mutation in the γ2-subunit in human heart results in a constitutively active form and has been linked to Wolff Parkinson White Syndrome which could lead to cardiomyopathy [29]. Although AMPK is known to phosphorylate a plethora of metabolic proteins, it is not currently known whether there is any isoform-specific association with a subset of known substrates. To enable the design of isoform-selective activators that might be required for activation of AMPK in specific tissues, it is critical to understand the biochemical properties and specific activities of different heterotrimers.

In this work, using enzyme kinetics, biochemical activation studies and phosphatase protection/modulation assay formats, we have probed the intrinsic enzyme activity of six recombinant AMPK isoforms and their functional activation by AMP and A769662. We found that α1-containing AMPK isoforms possess higher basal activity and are less sensitive to desphosphorylation by phosphatases compared with α2-containing heterotrimers. However, the α2-subunit-containing complexes are more readily activated by AMP than α1-complexes. We also measured the binding affinities of AMP, ADP and ATP using surface plasmon resonance (SPR) techniques which provide additional insights into allosteric regulation by these nts. A769662 selectively activates β1-containing AMPK isoforms, consistent with previous studies. Taken together, these studies provide a solid framework for the design and development of isoform-selective AMPK activators that are likely to be useful for the treatment of cardiovascular and metabolic diseases.

## EXPERIMENTAL

### Materials

Human recombinant protein phosphatase 2Aα catalytic subunit (PP2Aα) expressed in insect cells was purchased from Cayman Chemical. Biotinylated SAMS Peptide (Biotin-GGHMRSAMSGLHLVKRR-NH2) was purchased from ANASpec Inc. as a custom item. Rabbit anti-α-subunit-pThr^172/174^ antibody was purchased from Cell Signaling. Calmodulin from bovine brain was purchased from Sigma–Aldrich, okadaic acid was purchased from Calbiochem and multi-screen 384 phospho-cellulose filter plates were purchased from Millipore. [^33^P]ATP (3000 Ci/mmol) was purchased from PerkinElmer. AMPK activator, A769662, was purchased from R&D systems. All nts (AMP, ADP and ATP) were purchased from Sigma–Aldrich. The acetyl-CoA carboxylase (Ser^79^) SAMS peptide was purchased from Chinese Peptide Company and labelled in-house with Cy5 fluorofore. The Europium-labelled anti-phospho SAMS (Ser^79^) antibody was generated by custom labelling a Millipore mouse monoclonal pACC antibody with Europium Lantha screen Amine reactive chelate (Invitrogen).

### Cloning of AMPK constructs for bacterial expression

Recombinant full-length AMPK heterotrimeric complexes α1β1γ1, α1β2γ1, α1β2γ3, α2β1γ1, α2β2γ1 and α2β2γ3 were cloned into a tricistronic vector system as described previously [30]. A 6X-His tag was introduced at the N-terminal end of the α-subunit to facilitate purification. AMPK α1-kinase domain (α1-KD; residues 18–293) and α2-kinase domain (α2-KD; residues 7–282) were cloned into the pET28a vector (EDM Millipore) using standard molecular biology techniques. The α1-kinase and auto-inhibitory domain (α1-KD-AID) construct includes residues 18–348, and α2-kinase and auto-inhibitory domain (α2-KD-AID) construct includes residues 7–347. A 6X-His tag was introduced at the N-terminal end of the kinase domain to facilitate purification. To enable SPR studies, an AviTag™ sequence (GLNDIFEAQKIEWHE) was introduced at the N-terminal end of γ-subunit (α1β1γ1, α1β2γ1, α2β2γ1 and α2β2γ3) and co-expressed with biotin-ligase (BirA) in *E. coli*.

### Expression and purification of recombinant AMPK complexes

The constructs were transformed into *E. coli* BL21-CodonPlus™ (DE3)-RIPL strain (Agilent technologies) and transformants were selected on LB agar plates containing ampicillin (100 μg/ml). Single colonies were used to inoculate 10 ml of LB medium containing 100 μg/ml ampicillin. For large-scale expression and purification, an Erlenmeyer flask containing 1 litre of LB broth supplemented with 100 μg/ml ampicillin was inoculated with 25 ml of overnight culture and grown in a shaker incubator at 37°C to an *A*_600_ of 1.0. Protein expression was induced with 100 μM (final concentration) of isopropyl β-D-thiogalactopyranoside (IPTG), the temperature was reduced to 18°C and the cells were grown for an additional 18 h. Cells were harvested and resuspended in 50 ml lysis buffer [50 mM Tris, pH 8.0, 150 mM NaCl, 5% glycerol, 0.5 mM tris-2-carboxyethyl phosphine (TCEP), 25 mM imidazole and 0.001% Triton X-100]. The cell suspension was sonicated on ice with a Branson ultrasonic disintegrator (VWR Scientific Products) for 2–4 min at 50% duty cycle. Insoluble material was removed by centrifugation at 30,000 ***g*** in a Sorvall® RC5 plus centrifuge for 30 min at 4°C and the supernatant was loaded on to a 5 ml HisTrap™ HP column (GE Healthcare) and washed with five column volumes of lysis buffer. Bound proteins were eluted using a linear gradient (12 column volumes) with elution buffer (lysis buffer containing 300 mM imidazole). Fractions containing the expected protein were pooled based on SDS/10% PAGE analysis and dialysed overnight in dialysis buffer (50 mM Tris, pH 8.0, 150 mM NaCl, 10% glycerol, 0.5 mM TCEP and 0.001% Triton X-100).

### Activation of recombinant AMPK isoforms

The AMPK complexes and the kinase domains were activated by incubating 1.0 μM Ni^2+^-column purified sample in the presence of 200 nM CaMKKβ (The University of Dundee, Scotland, U.K.), in phosphorylation buffer (25 mM Tris, pH 7.5, 137 mM NaCl, 1 mM CaCl_2_, 5 mM MgCl_2_, 1 mM TCEP, 100 μM ATP and 200 nM calmodulin) for 30 min at 30°C in a thermostated shaker. The phosphorylated AMPK (pAMPK) complexes were re-purified on a HisTrap™ HP column as before, dialysed overnight and further purified by gel filtration chromatography using a Superdex 200 HiLoad 16/60 column (GE Healthcare) in SEC buffer (50 mM Tris, pH 8.0, 150 mM NaCl, 5% glycerol, 0.5 mM TCEP and 0.001% Triton X-100). The final product was stored at −20°C until further use. The protein concentrations were measured by absorbance at 280 using a NanoDrop 2000C spectrophotometer (Thermo Scientific).

### LC–MS

Direct analysis of purified AMPK protein intact mass measurements were conducted by LC–MS using a 0.5 mm × 100 mm Higgins Analytical C4 column (P/N RS-10M5-W045) and Waters Micromass LCT Premier mass spectrometer, as described before [30]. Chromatography was performed on an Agilent 1100 system operating at 0.020 ml/min. Solvents were: A, 0.1% formic acid; B, 0.1% formic acid in acetonitrile. Gradient steps were: 0–1 min, 2% B isocratic; 1–22.5 min, 2–75% B; 22.5–23 min, 75–100% B; 23–25.5 min, 100% B isocratic; 25.5–25.6 min, 100–2% B; 25.6–40 min, 2% B isocratic. LC–MS peptide mapping of AspN (Roche Applied Science) or trypsin (Sigma or Promega, both sequencing grade) digests of AMPK or its subunits was performed on a 0.5 mm × 100 mm Higgins Analytical C18 column (P/N RS-10M5-W185) and a Thermo Fisher LTQ XL ion trap mass spectrometer. Chromatography was performed on an Agilent 1100 system operating at 0.010 ml/min. Solvents were: A, 0.1% formic acid; B, 0.1% formic acid in acetonitrile. Gradient steps were: 0–2 min, 1.6% B isocratic; 2–100 min, 1.6–35% B; 100–110 min, 35–80% B; 110–111 min, 80–100% B; 111–120 min, 100% B isocratic; 120–121 min, 100–1.6% B; 121–140 min, 1.6% B isocratic. The instrument was programmed to acquire narrow-range (Zoom scan) and MS/MS spectra (collision energy setting 35) from the most intense peak in each survey scan, with peaks excluded for 30 s after a third MS/MS spectrum had been collected. Preliminary peptide identifications were made using Mascot software licensed from Matrix Sciences, and data were inspected and analysed directly using Xcalibur v. 2.2 (Thermo Fisher).

### Steady-state kinetic parameter determination for AMPK kinase isoforms

The kinase activity of pAMPK was measured via AMPK-mediated incorporation of ^33^phosphate from [^33^P]ATP into the synthetic peptide called SAMS [31] using the radiometric assay as described before [24]. This peptide has the amino acid sequence HMRSAM**S**GLHLVKRR and is derived from residues 73–85 of rat acetyl-CoA carboxylase in which Ser^77^ is mutated to alanine and the AMPK phosphorylation site is Ser^79^. Steady-state kinetic experiments to determine catalytic parameters were performed by varying one substrate while keeping the other fixed at 10-fold the *K*_m_ value. Assay linearity was ensured by determining that <30% substrate was depleted for all concentrations tested. The *K*_m_ values were obtained in the absence of AMP in the reaction. Data were fitted by non-linear regression to the Michaelis–Menten equation using GraphPad prism to obtain the kinetic parameters. The specific radioactivity of each isoform was determined at saturating concentrations of each substrate.

### TR-FRET intrinsic activity and allosteric activation assay

Intrinsic activity and allosteric activation of AMPK complexes by AMP and A769662 were determined using a TR-FRET-based kinase activity assay which utilized a Cy5-labelled SAMS peptide and a Europium-labelled phospho-ACC (Ser^79^) mouse monoclonal antibody. AMPK-mediated phosphorylation of the SAMS peptide allows antibody recognition and brings the donor and acceptor molecules in close proximity. After excitation at 320 nm, the energy from the Europium donor is transferred to the Cy5 acceptor which in turn generates light at 665 nm. The intensity of the signal is proportional to the level of AMPK-mediated Cy5 substrate phosphorylation. To assess the allosteric activation of AMPK complexes by AMP and A769662, the compounds were prepared in 100% DMSO and standard assay reactions contained 50 nM Cy5-SAMS peptide and ATP concentrations equal to the *K*_m_ for each isoform complex as described before [30]. The AC_50_ values (estimated concentration of activator required to reach half-maximal phosphorylation of SAMS peptide) were calculated using GraphPad Prism software. Non-specific emission obtained from samples containing all the reagents except enzyme was used to normalize the values.

Intrinsic kinase activity of different AMPK isoforms was measured using 50 nM Cy5-SAMS and 20 μM ATP and EC_50_ values [effective concentration of AMPK required for half maximal (50%) phosphorylation of SAMS peptide in assay condition] were calculated using GraphPad Prism software.

### Protection of pAMPK complexes from phosphatase by slot-blot ELISA

To measure the effect of AMP and A769662 on PP2Aα-catalysed dephosphorylation of pAMPK α-pThr^172/174^, fully phosphorylated AMPK was pre-incubated with test compounds in assay buffer containing 50 mM HEPES, 10 mM MgCl_2_, 1 mM EGTA, 0.01% Tween-20 and 0.01% BSA at pH 7.4. Test compound was previously diluted in half-log increments, starting at 40 μM in 100% DMSO, diluted 10-fold in assay buffer and dispensed into a microtitre plate. After 15 min at room temperature, phosphatase was added to the mixture and the reaction was allowed to proceed for 60 min at room temperature. Reactions were quenched by the addition of okadaic acid, and transferred to PVDF membrane using the slot-blot apparatus and subjected to immunoblotting. Phosphatase sensitivity was similarly assessed by subjecting pAMPK to a phosphatase (PP2Aα) treatment using various concentrations and incubation times. The concentration of phosphatase was expressed as nmol of PP2Aα per minute per nmol of pAMPK in the reaction.

### Immunoblotting

The membrane was blocked with Western Blot Blocking Buffer Reagent (Li-Cor) for 3–4 h at room temperature. The blot was probed using a rabbit α-pThr^172/174^-specific antibody (Cell Signaling 40H9) at 1:500 dilution for 2 h and washed three times using 1 × PBS/0.1% Tween 20 (42°C pre-warmed wash buffer). Following the wash step, goat anti-rabbit 800CW (Rockland) at 1:10000 dilution was used as a secondary antibody. Blots were visualized using the Li-Cor Odyssey infrared imaging system and signal quantification was performed using the Odyssey software. The pEC_50_ values (estimated concentration of activator required for half-maximal protection of pThr^172/174^ from PP2α) were calculated using GraphPad Prism software.

### Surface plasmon resonance experiments

SPR experiments were performed on a Biacore™ 3000 instrument (GE Healthcare). BAP-tagged AMPK isoform (α1β1γ1, α1β2γ1, α2β2γ1, α2β2γ3) was captured on to a streptavidin sensor chip to levels ranging from 4000 to 6000 RU. Binding experiments were performed in 25 mM Tris, pH 7.5, 150 mM NaCl, 250 μM TCEP, 0.01% P20, 0.5 mg/mL BSA, 2% DMSO ± 25 nM staurosporine (Stau) (Roche) and ±150 μM AMP at 25°C. Binding responses were processed using Scrubber 2 (BioLogic Software Pty Ltd) to zero, x-align, double reference and corrected for excluded volume effect of DMSO in the data. To determine binding affinities of compound A769662, at least four concentrations with 3-fold dilutions were tested in duplicate. The compound was injected at a flow rate of 50 μl/min with total contact time of 120 s and dissociation time of 600 s. Rate parameters (*k*_on_, *k*_off_) and corresponding dissociation constant(*K*_D_=*k*_off_/*k*_on_) were determined by globally fitting all of the experimental data to a simple 1:1 interaction model using Biaeval software (GE Healthcare). To determine nt binding, 10 concentrations with 2-fold dilutions were tested in duplicate. The highest concentration tested was 2 mM. The compound was injected at a flow rate of 50 μl/min with total contact time of 120 s and dissociation time of 300 s. Equilibrium dissociation constant (*K*_D_) was determined using a steady-state affinity algorithm available within Scrubber.

## RESULTS

### Expression, purification and activity of AMPK isoforms and kinase domains

**Figure 1:**
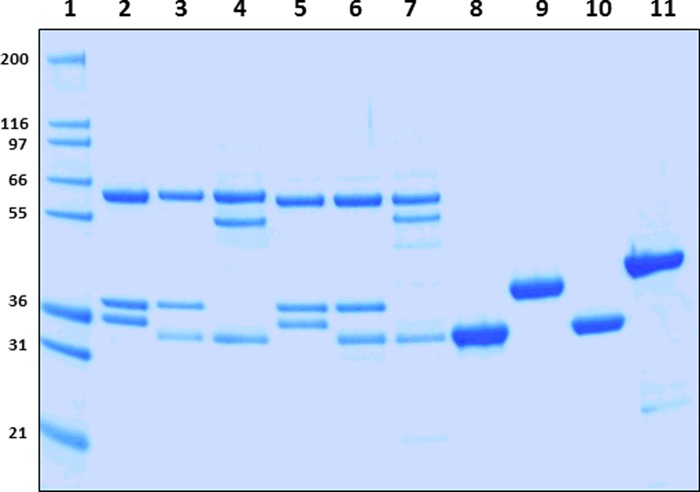
Analysis of recombinant AMPK protein reagents Coomassie-stained SDS/10% PAGE analysis of the activated and purified AMPK proteins. *Lane 1*, molecular mass protein marker (Marker 12, Invitrogen); *lane 2*, AMPK-α1β1γ1; *lane 3*, AMPK-α1β2γ1; *lane 4*, AMPK-α1β2γ3; *lane 5*, AMPK-α2β1γ1; *lane 6*, AMPK-α2β2γ1; *lane 7*, AMPK-α2β2γ3; *lane 8*, AMPK-α1-KD; *lane 9*, AMPK-α1-KD+AID; *lane 10*, AMPK-α2-KD; *lane 11*, AMPK-α2-KD+AID. Mass of the protein markers (in kDa) shown on the left. Each lane contained 2.0 μg of recombinant protein.

**Figure 2:**
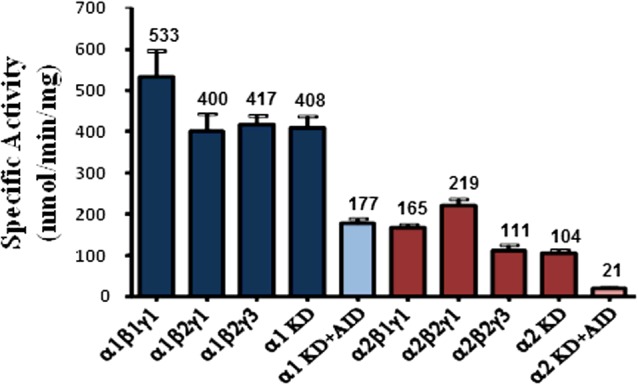
Specific activities of α1- and α2-containing AMPK complexes Specific activities were measured using the radiometric assay in the absence of AMP. The results are expressed as mean ± S.E.M. for three replicate assays.

Using the tricistronic expression system, we have expressed 6 out of a possible 12 AMPK isoforms in *E. coli*. The AMPK isoforms were purified using Ni^2+^-affinity and size exclusion chromatography to >90% purity, as assessed by SDS/PAGE and MS analysis. The yields of the full-length AMPK heterotrimeric complexes were ∼2–3 mg/l except the kinase domains which yielded >6.0 mg/l. As shown in Figure 1, we detected the expected molecular masses of the subunits (α, β, γ and kinase domains) at stoichiometric levels even though they were captured via the His-tag present only on their α-subunits. Activation of the AMPK complexes was achieved by treating with CaMKKβ and the extent of α-Thr^172/174^ phosphorylation was estimated by MS analysis. As shown in Table 1, we obtained ∼88–99% phosphorylation at α-Thr^172/174^ site. In general, the specific activity of the α1-complexes and α1-KD was ∼3-fold greater than the α2-complexes or α2-kinase domain (Figure 2). Protein reagents that contain both kinase domain and the auto-inhibitory domain residues (KD+AID) have reduced (2.5-fold for α1 and 5.0-fold for α2) specific activity (Figure 2). We have also determined the effective concentration of the AMPK isoforms required for the half-maximal phosphorylation (EC_50_) of the substrate peptide (SAMS). As shown in Table 1 (intrinsic kinase activity), the EC_50_ values for α1-isoforms were 10- to 20-fold lower than for the α2-isoforms.

**Table 1:** Enzymatic activity, phosphatase sensitivity and kinetics of α1- and α2-containing AMPK isoforms ND, not determined. *dEC_50_ (effective concentration of PP2a required for the half-maximal dephosphorylation of AMPK isoforms in the absence of activator) values were determined from slopes of Figure 6. ^†^Kinase activity of recombinant AMPK isoforms treated with CaMKKβ was measured by SAMS assay in the presence of 20 mM ATP as described before. Although the concentration of ATP and SAMS peptide were not individually optimized for the different AMPK isoforms, these activity values nevertheless show the general trend of the intrinsic kinase activity of the different heterotrimeric AMPK complexes. EC_50_ values represent the effective concentration of the enzyme required for the half-maximal phosphorylation of SAMS peptide. ^‡^Percentage of α-pThr^172/174^ phosphorylated by CaMKKβ as measured by MS. ^§^Kinetic parameters were obtained in the absence of AMP.

				*K*_m_ (μM)^§^	
AMPK isoforms	PP2Aα sensitivity dEC_50_ (nM)*	Intrinsic kinase activity EC_50_ (nM)^†^	pThr^172/174‡^ (%)	ATP	SAMS
α1β1γ1	1145±692	0.38±0.09	93–97	26.04±5.6	26.67±3.1
α1β2γ1	1966±207	0.46±0.17	87–94	45.06±5.8	32.27±3.5
α1β2γ3	441.3±307	0.49±0.04	84–88	332.9±65	37.31±3.9
α1-KD	3.211±0.294	2.89±0.58	92–94	ND	ND
α1-KD+AID	ND	53.2±6.68	ND	ND	ND
α2β1γ1	39.87±4.065	4.37±1.37	95–99	31.35±9.3	121.4±13
α2β2γ1	56.84±26.00	1.73±0.54	92–94	47.97±5.0	107.6±7.1
α2β2γ3	40.70±4.603	10.13±3.54	86–92	416.8±39	80.03±13
α2-KD	1.293±0.024	>240	94–96	ND	ND
α2-KD+AID	ND	>240	ND	ND	ND

### Steady-state kinetic studies

The steady-state kinetic parameters for different AMPK isoforms were determined by varying the concentration of one of the substrates while keeping the other fixed at ∼10-fold the *K*_m_ value. The apparent Michaelis–Menten constants (*K*_m_) values for SAMS peptide at fixed ATP concentration were between 26 and 37 μM for α1-complexes whereas it was between 80 and 121 μM for α2-complexes (Table 1). The *K*_m_ values for ATP were comparable between α1- and α2-isoforms however it varied depending on the γ-isoform. For AMPK complexes that contain the γ3-subunit, the estimated *K*_m_ values of ATP were 332.9±65 μM and 416.9±39 μM for α1β2γ3 and α2β2γ3 respectively (Table 1), which is ∼10-fold higher than that observed for γ1-containing complexes (26.04±5.6, 45.06±5.8, 31.35±9.3 and 47.97±5.0 μM for α1β1γ1, α1β2γ1, α2β1γ1 and α2β2γ1 respectively).

### SPR nt binding studies

**Figure 3:**
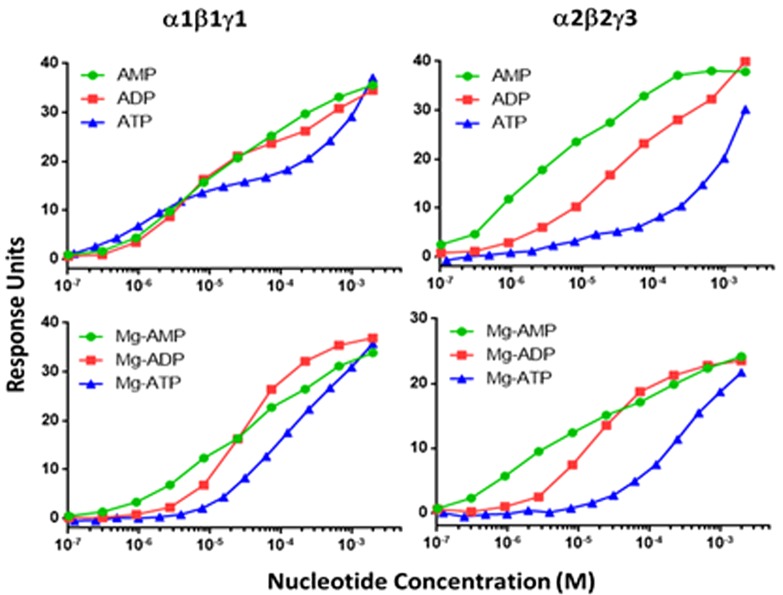
Comparison of nt binding to α1β1γ1 and α2β2γ3 complexes in the presence and absence of Mg^2+^using SPR techniques A representative dose-dependent SPR response run on nt binding to α1β1γ1 and α2β2γ3 is shown. Data are mean (*n*=3), and curves were generated using best-fit values. The binding affinities were calculated using a steady-state affinity algorithm. The *K*_D_ values calculated from the curves are shown in Table 2.

We then compared the binding of nts (AMP, ADP and ATP) to α1β1γ1, α1β2γ1, α2β2γ1 and α2β2γ3 isoforms in the presence and absence of Mg^2+^ and Stau, using SPR technique. As shown in Figure 3, the nts displayed a biphasic binding curve with two distinct binding affinities (*K*_D1_ and *K*_D2_) in the absence of Mg^2+^. However, in the presence of either Mg^2+^ or Mg^2+^ and Stau, ADP and ATP binding curves, but not AMP affinity curves, were altered from biphasic to a monophasic curve. As shown in Table 2, the *K*_D_ value of the tighter binding site (*K*_D1_) for AMP was between 1 and 4 μM for all the isoforms irrespective of the presence or absence of Mg^2+^ and Stau. The second weaker binding site (*K*_D2_) for AMP showed a significantly reduced affinity and the values were 260±30, 340±70, 560±90 and 42±4 μM for α1β1γ1, α1β2γ1, α2β2γ1 and α2β2γ3 respectively (Table 2). Addition of stoichiometric excess of Mg^2+^, with or without Stau, did not alter the binding affinity of AMP for the tighter binding site (*K*_D1_). The weaker binding site of the two (*K*_D2_), however, showed a 3-fold improvement in binding for α1β1γ1, α1β2γ1 and α2β2γ1 complexes in the presence of Mg^2+^ and Stau as compared with Mg^2+^ alone; the respective *K*_D2_ values were 70±10, 130±20 and 70±10 μM (Table 2). Interestingly, the α2β2γ3 complex maintained similar binding affinity for AMP at the weaker site (*K*_D2_) in the presence and absence of Stau (∼40 μM).

**Table 2:** Nucleotide binding kinetics determined by SPR BAP-tagged AMPK isoforms were captured on to a streptavidin sensor chip to levels ranging from 4000 to 6000 RU. The compounds were injected at a flow rate of 50 μl/min with total contact time of 120 s and dissociation time of 600 s. Rate parameters (*k*_on_, *k*_off_) and corresponding dissociation constant(*K*_D_=*k*_off_/*k*_on_) were determined by globally fitting all of the experimental data to a simple 1:1 interaction model using Biaeval software (GE Healthcare).

	α1β1γ1		α1β2γ1		α2β2γ1		α2β2γ3	
nts	*K*_D1_ (μM)	*K*_D2_ (μM)	*K*_D1_ (μM)	*K*_D2_ (μM)	*K*_D1_ (μM)	*K*_D2_ (μM)	*K*_D1_ (μM)	*K*_D2_ (μM)
AMP	3.7±0.2	260±30	4.3±0.3	340±70	2.9±0.2	560±90	1.6±0.2	42±4
ADP	4.75±0.08	1010±80	4.2±0.2	2600±400	2.47±0.09	2600±300	11.2±0.3	1500±200
ATP	1.35±0.02	2260±90	1.10±0.04	3400±300	0.92±0.03	2900±200	7.6±0.6	3500±300
Mg-AMP	4.50±0.3	180±20	1.83±0.06	330±20	1.50±0.1	230±20	1.77±0.06	150±10
Mg-ADP	33.3±0.3		28.2±0.3		22.8±0.5		19.0±0.2	
Mg-ATP	130±1		95±1		95±1		277±4	
Mg-AMP+Stau	2.4±0.2	70±10	0.90±0.10	130±20	1.50±0.10	70±10	1.60±0.20	43±7
Mg-ADP+Stau	24.4±0.4		15.0±1.0		11.9±0.3		12.5±0.1	
Mg-ATP+Stau	300±10		400±20		133±4		540±20	

ADP showed two distinct binding affinities in the absence of Mg^2+^ and Stau and the *K*_D1_ values of the tighter binding site were 4.75±0.08, 4.2±0.2, 2.47±0.09 and 11.2±0.3 μM for α1β1γ1, α1β2γ1, α2β2γ1 and α2β2γ3 respectively. The *K*_D2_ values for the weaker binding site for ADP were 1010±80, 2600±400, 2600±300 and 1500±200 μM for α1β1γ1, α1β2γ1, α2β2γ1 and α2β2γ3 respectively. The Mg-ADP showed a single measurable binding affinity and the *K*_D_ values were 33.3±0.3, 28.2±0.3, 22.8±0.5 and 19±0.2 μM for α1β1γ1, α1β2γ1, α2β2γ1 and α2β2γ3 respectively. The ADP *K*_D_ values in the presence of either Mg^2+^ or Mg^2+^ and Stau were comparable (Table 2).

ATP also showed two distinct binding affinities in the absence of Mg^2+^ and Stau and the *K*_D1_ values of the tighter binding site were 1.35±0.02, 1.10±0.04, 0.92±0.03 and 7.6±0.6 μM for α1β1γ1, α1β2γ1, α2β2γ1 and α2β2γ3 respectively. The ATP binding affinity for the weaker binding site was >1000-fold weaker than the tighter binding site and the estimated *K*_D2_ values were 2260±90, 3400±300, 2900±200 and 3500±300 μM for α1β1γ1, α1β2γ1, α2β2γ1 and α2β2γ3 respectively. The Mg-ATP showed a single measurable binding affinity and the *K*_D_ values were 130±1.0, 95±1.0, 95±1.0 and 277±4.0 μM for α1β1γ1, α1β2γ1, α2β2γ1 and α2β2γ3 respectively. The ATP *K*_D_ values in the presence of both Mg^2+^ and Stau were not significantly different from that of Mg-ATP (Table 2).

### Modulation of activity of AMPK isoforms by A769662 and AMP

**Figure 4:**
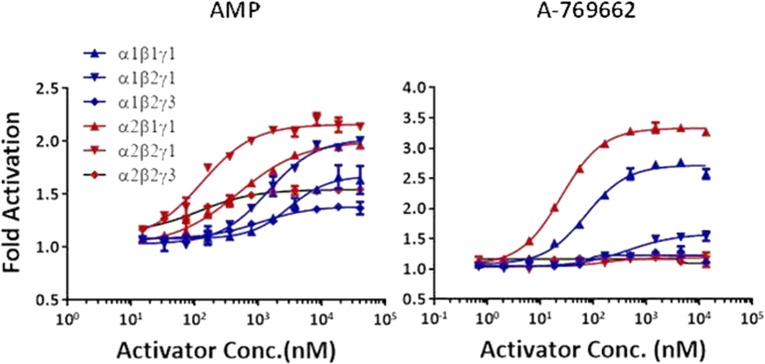
Effect of allosteric activation by AMP and A769662 Dose-dependent activation of AMPK complexes was measured by TR-FRET assay as described in the ‘Experimental’ section. The AC_50_ (concentration of the activators required for the half-maximal activation of the AMPK isoforms) values are presented in Table 3. Data are mean (*n*=2), and curves were generated using best-fit values.

**Figure 5:**
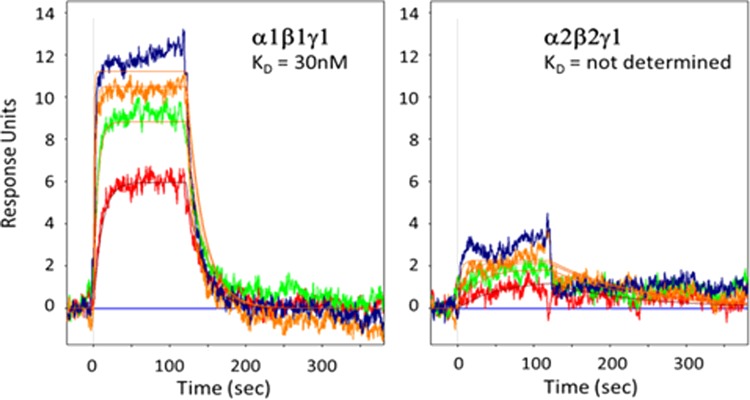
SPR binding response of A769662 to α1β1γ1 and α2β2γ1 Dose-dependent (1000, 333, 111 and 37 nM) binding of A769662 to AMPK isoforms in the presence of 150 μM AMP. Rate parameters (*k*_on_, *k*_off_) and corresponding dissociation constant (*K*_D_=*k*_off_/*k*_on_) were determined by globally fitting using Biaeval software. Equilibrium dissociation constant (*K*_D_) was determined using a steady-state affinity algorithm available within Scrubber.

The allosteric modulation of AMPK activity was studied at increasing concentrations of the activator (AMP and A769662) while keeping fixed enzyme and SAMS concentration and setting the ATP concentration at the *K*_m_ for each AMPK complex. The basal kinase activity of the pAMPK isoforms in the absence of activator was normalized to zero (baseline) and the changes in the enzyme activity in the presence of activator at different concentrations was compared with the basal activity. The AC_50_ values (activator concentration required for the half-maximal phosphorylation of SAMS peptide) were determined as described in ‘Experimental’ section. As shown in Table 3, AMP increases the catalytic activity of all the six isoforms 1.3- to 2-fold relative to their intrinsic activities. In general, the α2-containing isoforms was more readily activated than α1-containing isoforms as shown in Figure 4. The AMP concentration required to achieve the maximal activation of α2-containing isoforms was ∼7-fold less than that of α1-containing isoforms (Table 3 and Figure 4). The estimated AC_50_ values (Table 3) of AMP for α1β1γ1, α1β2γ1 and α1β2γ3 were 3064, 1415 and 1042 nM respectively, but significantly left-shifted for α2β1γ1 (478 nM), α2β2γ1 (129 nM) and α2β2γ3 (117 nM). As expected, A769662, activates β1-subunit containing isoforms (α1β1γ1 and α2β1γ1) ∼3-fold, but showed only a modest effect (<1.2-fold) on the β2-containing isoforms (Figure 4). The estimated AC_50_ values of A769662 for α1β1γ1 and α2β1γ1 were 72.24 and 24.68 nM respectively, whereas the AC_50_ values for β2-subunit-containing isoforms were >40 μM (Table 3). We also measured the direct binding of A769662 to BAP-tagged β1- and β2-containing isoforms (α1β1γ1 and α2β2γ1) in the presence of 150 μM AMP using SPR techniques. As shown in Figure 5, A769662 binds to α1β1γ1in a dose-dependent manner whereas the binding to α2β2γ1 was negligible. The estimated *K*_D_ (*n*=1) for A769662 to α1β1γ1 was 30 nM (Figure 5), which is comparable (48 nM) to what we have reported previously [24].

**Table 3:** Phosphatase sensitivity, allosteric activation and protection of AMPK isoforms *AC_50_ (concentration of the activators required for the half-maximal activation of the AMPK isoforms) values were determined from the slopes of Figure 4. ^†^pEC_50_ (effective concentration of the activator required for the half-maximal protection of pThr^172/174^ from dephosphorylation) values were determined from the slopes of Figure 7.

	Activation AC_50_ (nM)*		Fold activation		Protection pEC_50_ (nM)^†^	
AMPK isoforms	AMP	A769662	AMP	A769662	AMP	A769662
α1β1γ1	3064	72.24	1.67	2.71	500	46
α1β2γ1	1415	>40000	2.02	1.58	300	4700
α1β2g3	1042	>40000	1.38	1.22	454	>40000
α2β1γ1	478	24.68	2.0	3.33	112	40
α2β2γ1	129.2	>40000	2.16	1.18	42	2324
α2β2γ3	116.8	>40000	1.54	1.67	139	5600

### Comparison of phosphatase sensitivity of α1- and α2-containing isoforms

**Figure 6:**
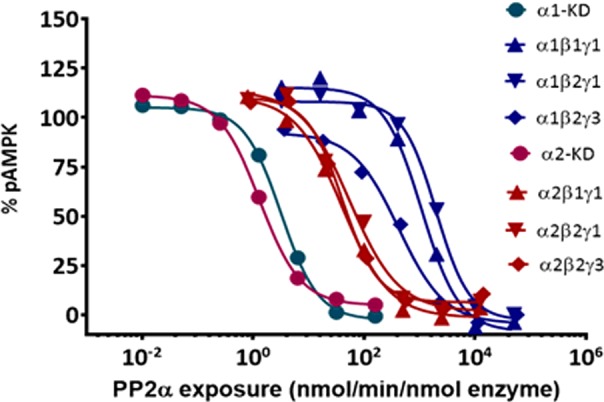
Phosphatase (PP2Aα) sensitivity of AMPK isoforms Effect of phosphatase (PP2Aα) on pThr^172/174^ dephosphorylation of AMPK isoforms in the absence of activators is shown. Data are mean (*n*=2), and curves were generated using best-fit values. The dEC_50_ (effective concentration of PP2Aα required for the half-maximal dephosphorylation) values are presented in Table 1.

We then examined the sensitivity of phospho-α-Thr^172/174^ (pThr^172/174^) for dephosphorylation by phosphatase (PP2Aα) in the absence of allosteric modulators. The extent of dephosphorylation was measured using an antibody specific to α-pThr^172/174^ as described in ‘Experimental’ section. Results showed that isolated kinase domains were more sensitive to dephosphorylation by PP2Aα  as compared with the heterotrimeric complexes and the estimated PP2Aα concentrations required for the half maximal dephosphorylation (dEC_50_) were 3.2 and 1.3 nM for isolated α1- and α2-kinase domains respectively (Table 1). The presence of the regulatory subunits (β and γ) leads to increased protection of pAMPK from dephosphorylation compared with the kinase domains alone (Figure 6). As shown in Table 1, the dEC_50_ values for α1-containing isoforms were 1145, 1966 and 441 nM for α1β1γ1, α1β2γ1 and α1β2γ3 respectively; ∼100- to 600-fold right-shifted compared with that for isolated α1-kinase domain. The dEC_50_ values for α2-containing isoforms were 40, 57 and 41 nM for α2β1γ1, α2β2γ1 and α2β2γ3 respectively; 13- to 18-fold right-shifted compared with that for isolated α2-kinase domain (Figure 6). These data suggested that the α2-containing isoforms were significantly (>25-fold) more sensitive to PP2Aα than α1-containing isoforms.

### Allosteric modulators mediated protection of α-pThr^172/174^ from phosphatase

**Figure 7:**
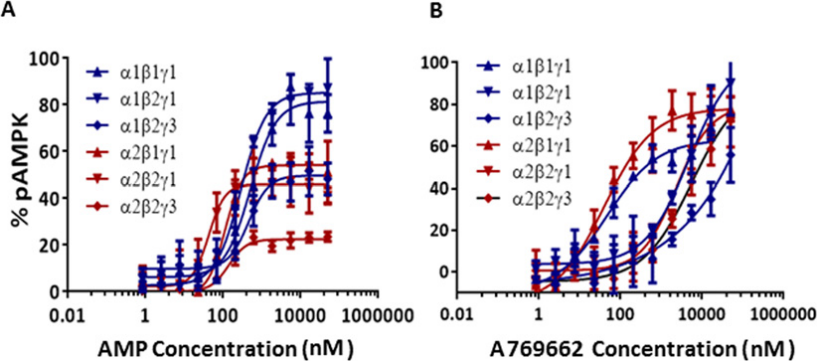
Allosteric activators on protection of pThr^172/174^ from phosphatase (PP2Aα) Protection against dephosphorylation of α-pThr^172/174^ was determined by incubating 10 nM of AMPK with 40 nM of PP2Aα in the presence of increasing concentrations of activator and the amount of dephosphorylation was measured by immunoblotting. Data are mean (*n*=2), and curves were generated using best-fit values. The band intensities were converted and expressed as percentage of α-pThr^172/174^ protected from dephosphorylation. The pEC_50_ (effective concentration of activator required for the half-maximal protection from dephosphorylation) values are presented in Table 3.

To study the allosteric modulation and protection of α-pThr^172/174^ from phosphatase, we incubated the AMPK complexes with various concentrations of allosteric activator (AMP or A769662) and then treated with fixed concentration of phosphatase as described in the ‘Experimental’ section. The amount of α-pThr^172/174^ protected from PP2Aα was measured using an antibody specific to α-pThr^172/174^. Although A769662 provided >70% protection for all the isoforms studied, the protection of β2-isoforms was evident only at relatively high concentrations (Figure 7). The pEC_50_ (concentration required for half-maximal protection of α-pThr^172/174^ from PP2Aα) values of A769662 for β1-containing complexes were 46 and 40 nM for α1β1γ1 and α2β1γ1 respectively. As was observed with the allosteric activation assay, the β2-containing complexes were significantly right-shifted and the pEC_50_ values for α1β2γ1, α1β2γ3, α2β2γ1 and α2β2γ3 were 4700, >40,000, 2324 and 5600 nM respectively (Table 3). AMP showed >80% protection for α1β1γ1, α1β2γ1, ∼50% protection for α1β2γ3, α2β1γ1, α2β2γ1 and only ∼20% protection for α2β2γ3 (Figure 7). The pEC_50_ values for AMP were 500, 300 and 454 nM for α1β1γ1, α1β2γ1 and α1β2γ3 respectively, and were 112, 42 and 139 nM for α2β1γ1, α2β2γ1 and α2β2γ3 respectively (Table 3).

## DISCUSSION

AMPK is a serine/threonine kinase that plays a central role in regulating lipid metabolism and energy homoeostasis at cellular and tissue level that makes it an attractive target for therapeutic intervention for diseases such as diabetes and obesity. Several pharmacological AMPK activators have shown beneficial outcomes in animal studies [16]. Though AMPK heterotrimers are ubiquitously expressed, studies show significant difference in the expression pattern between species and between different cell types. For example, AMPK α1β2γ1 is predominantly expressed in human liver cells whereas AMPK α2β1γ1 is predominant in rodent liver cells, γ3 is expressed exclusively in muscle cells and γ2 is expressed in heart tissue. The activation mechanism and functional significance of these diverse isoforms in tissues are poorly understood. In the present study, we have generated six recombinant AMPK isoforms which include three α1-containing complexes (α1β1γ1, α1β2γ1 and α1β2γ3) and three α2-containing complexes (α2β1γ1, α2β2γ1 and α2β2γ3) and compared their specific activity, substrate binding kinetics and magnitudes of activation and protection from dephosphorylation by phosphatase (PP2Aα) by the endogenous ligand, AMP and the synthetic ligand, A769662.

The recombinant AMPK complexes were expressed in *E. coli*, purified and validated by SDS/PAGE and MS techniques. The reagents were purified to >90% purity as determined by MS and gel analysis (Figure 1). The enzymatic activity of the un-activated *E. coli* expressed recombinant AMPK isoforms was insignificant in our assays (results not shown) therefore no further experiments were performed with un-activated AMPK reagents. Activation of the AMPK complexes was achieved by treating with an upstream kinase, CaMKKβ, which phosphorylates AMPK specifically at α-Thr^172/174^ (Thr^172^ in α1 and Thr^174^ in α2). Previous reports have indicated that phosphorylation of α-Thr^172/174^ (pThr^172/174^) significantly improves (500- to 1000-fold) the intrinsic activity (basic kinase activity) of AMPK isoforms [24,30]. Since the extents of phosphorylation at α-Thr^172/174^ directly influence the enzymatic activity, we first measured α-pThr^172/174^ levels using quantitative mass spectrometric evaluation of peptides bearing the phospho-threonine motifs. Our data showed 88–99% of α-Thr^172/174^ phosphorylation among different isoforms suggesting they are more or less fully phosphorylated on their activation loops (Table 1).

We then measured the specific activities of a panel of activated (phosphorylated) AMPK isoforms by monitoring the AMPK-mediated incorporation of ^33^phosphate from ^33^P[ATP] into the SAMS peptide at saturating concentrations of substrates (ATP and SAMS peptide). Results showed that the specific kinase activities of α1-complexes (α1β1γ1, α1β2γ1, α1β2γ3) were ∼3-fold higher (Figure 2) than that of α2-complexes (α2β1γ1, α2β2γ1, α2β2γ3). Similar results were obtained for the isolated α1- and α2-kinase domains suggesting that the kinase domain in the AMPK α1-subunit is intrinsically more active than the α2 kinase domain. These results imply that although human α1- and α2-subunits share 90% amino acid sequence identity within the catalytic core (∼77% overall sequence identity), there are subtle differences in their basal kinase activities. Interestingly, there is a significant reduction (2.3- and 4.9-fold for α1- and α2-kinase domain respectively) in the kinase activity when the AID region was appended to the kinase domain (KD-AID). These results are consistent with the previous reports on yeast AMPK homologue (*Saccharomyces cerevisiae*) [23]. Crystal structures of KD-AID constructs of yeast as well as mammalian AMPK show that productive interaction of the AID domain with the critical C-helix of the kinase module leads to a low-activity conformation [23,32]. In the activated full-length AMPK structures that have been reported to date, the AID domain is either completely disordered [24] or appears to dock on the γ-subunit [25,32].

Next we measured the *K*_m_ values for the substrates, ATP and SAMS peptide, by steady-state kinetic experiments. As shown in Table 1, the α1-complexes exhibited ∼3-fold lower *K*_m_ for SAMS peptide than the corresponding α2-complexes which is consistent with the specific activities of the AMPK reagents. On the other hand, the ATP *K*_m_ values were comparable for all the isoforms (α1- and α2-complexes) except those that contain γ3-subunit (α1β2γ3 and α2β2γ3). The estimated ATP *K*_m_ values for α1β2γ3 and α2β2γ3 were ∼4-fold and ∼10-fold higher respectively, compared with γ1-isoforms. These results are somewhat surprising since the conserved portion of the γ-subunits that contain the CBS 1–4 modules is located ∼50 Å (1 Å=0.1 nm) away from the ATP-binding site in the kinase module. The γ2- and γ3-subunits contain large, non-conserved N-terminal extensions upstream of the common nt-binding CBS motifs in the γ-subunits and little is known about their folding or functional significance. One possible explanation for higher ATP *K*_m_ values for the γ3-containing isoforms could be due to the direct interaction of the N-terminal extension in γ3 with the kinase domain, effecting binding of ATP at the kinase hinge region or the peptide substrate either directly or indirectly.

The crystal structures of mammalian AMPK complexes have revealed that ATP could also bind at the nt-binding sites on the γ-regulatory subunits in addition to the α-kinase domain. At the catalytic α-subunit, ATP functions as a co-substrate for kinase reaction whereas at the γ-subunit it functions as an allosteric antagonist of AMPK activation by AMP. Using SPR techniques, we measured the binding of ATP, ADP and AMP to α1β1γ1, α1β2γ1, α2β2γ1 and α2β2γ3 isoforms in the presence and absence of Mg^2+^ and a known kinase inhibitor, Stau. The *K*_D_ values derived in the presence of Stau essentially represent the binding constants for the nts at the γ-subunit, as the binding to α-subunit is blocked. In contrast, the *K*_D_ values obtained in the absence of Stau are a composite of binding to both α- and γ-subunits. Our results showed two distinct binding events (biphasic binding curve) for all the three nts (AMP, ADP and ATP) with significantly different affinities and are comparable between the isoforms. Equilibrium dissociation constants of binding of AMP to the tighter of the two binding sites are ∼1.0–7 μM whereas they were right-shifted ∼100× for the weaker binding site (Figure 3). Interestingly, AMP appears to have stronger binding affinity to site 3 of γ3 (∼40 μM) than γ1 (∼300–600 μM). Compared with AMP, the binding affinities for ADP and ATP are significantly weaker (in the mM range) for the second site. High resolution crystal structures of truncated AMPK regulatory core have revealed binding of AMP at three of the four possible nt-binding sites of γ1-subunit [22,32]. Of these, site 4 is a non-exchangeable or slowly-exchangeable AMP site that appears to serve a structural rather than a functional role [22].

Previously, Gamblin and co-workers have reported equilibrium dissociation constants for the binding of various nts to full-length, pAMPK based on fluorescence competition experiments with NADH or coumarin adducts with ADP or ATP [22]. These studies revealed that all the three nts (AMP, ADP and ATP) bound to AMPK at least at two distinct sites. Based on the quenching of fluorescence of coumarin-AXPs, they estimated equilibrium dissociation constants of 1.0–2.0 μM for the stronger site and 50–80 μM for the weaker site for all the three nts. However, the presence of Mg^2+^ resulted in weaker interaction of ATP to both the stronger binding site (18 μM) and the weaker binding site (230 μM). Since they used full-length AMPK for their studies, one caveat is that it is difficult to precisely estimate the relative contribution of the α-subunit kinase module and the γ-subunit for the reported binding affinities. Additional crystallographic studies suggested that site 1 on the γ-subunit is the tighter binding site and site 3 is the weaker binding site. Our SPR binding studies for the various nts are generally consistent with the previous studies and show equilibrium dissociation constants of 1–7 μM for site 1 and 300–3000 μM for site 3. Although the affinity of AMP for the tighter binding site is similar between γ1- and γ3-containing isoforms (1.6 to 4.3 μM), the weaker site in γ1 appears to be less competent to bind AMP (260–560 μM) compared with γ3 (42 μM).

Given that under physiological conditions, most of the cellular ATP is coordinated to Mg^2+^, we also measured the nt binding in the presence of Mg^2+^. Results in Table 2 showed that Mg-ATP yielded a single measurable *K*_D_ value ranging from 100 to 300 μM which is ∼100-fold weaker than *K*_D1_ and ∼10-fold stronger than *K*_D2_ obtained with free ATP. The single binding constant obtained for each of the AMPK isoforms with Mg-ATP is a composite of the nt binding to the ATP site of kinase (α-subunit) and the regulatory sites of γ-subunit. Most likely, these binding affinities are comparable in magnitude such that the deconvolution algorithm used for SPR data analysis is unable to differentiate between them. Our data clearly indicate that Mg^2+^ significantly attenuates the affinity of ATP for the tight exchangeable site in the γ-subunit as previously reported [22]. Interestingly, the binding of Mg-ADP was also altered from a biphasic binding curve to a single binding mode with *K*_D_ values ranging from 18 to 32 μM suggesting an equivalent effect similar to Mg-ATP. This might not be relevant *in vivo* as the vast majority of ADP is not coordinated with Mg^2+^ under physiological conditions. As expected, Mg-AMP retained similar binding affinities to AMP for both γ1- and γ3-containing isoforms.

In the presence of Stau, a potent kinase inhibitor that more or less completely blocks the ATP site of most kinases, the binding affinities of Mg-ADP are similar to those observed in its absence. In contrast, the binding affinities for Mg-ATP get attenuated 2- to 4-fold by Stau. These competition SPR experiments with Stau suggest that Mg-ATP binds to the γ-subunit of different AMPK isoforms with equilibrium dissociation constants in the 100–500 μM range, ∼100-fold weaker than free ATP. Previous studies using fluorescent ATP analogues had shown that Mg-ATP binds to pAMPK α1β1γ1 up to 10-fold weaker than free ATP [22]. The slight discrepancies between these values could be due to the different experimental protocols that have been used. Nevertheless, these studies conclusively demonstrate that the ATP-binding sites on the γ-subunits of AMPK are less tolerant of Mg-ATP compared with free ATP. In contrast, chelation with divalent metal ions (Mg^2+^ or Mn^2+^) is absolutely required for binding of ATP to the kinase domain and its catalytic activity.

Stau appears to enhance the binding affinities of AMP to γ1-containing isoforms but not to AMPK α2β2γ3. Although the effect is only modest for the stronger binding site on the γ-subunit and within experimental error, the affinity of AMP for the weaker binding site is augmented by ∼3- to 8-fold by Stau. It is unknown whether binding of ATP to the kinase module would elicit a similar response. Nevertheless, these results suggest an intriguing possibility of a two-way communication between the kinase domain and site 3 of the γ-subunit. Binding of a ligand at the kinase ATP site enhances the binding affinity of AMP for site 3 whereas AMP binding at the γ-subunit leads to allosteric conformational changes on the α-subunit and consequent effects in its functional activity.

We next studied modulation of different AMPK isoforms by small molecules in two distinct assay formats: intrinsic activation and protection from dephosphorylation by phosphatases. The intrinsic activation assay measures the degree to which a small molecule modulator increases the catalytic activity of pAMPK as measured by the phosphorylation of a peptide (SAMS) substrate. Unless otherwise stated, all experiments were performed at fixed concentrations of peptide substrate and ATP at *K*_m_ values, with the allosteric effect being a composite of influences on both *V*_max_ and *K*_m_. The phosphatase protection assay measures the degree to which a small molecule modulator induces a conformation in pAMPK complex which occludes access of a phosphatase (PP2Aα) to the α-subunit residue pThr^172/174^ that is essential for catalytic activity. Our results showed that although the α1-complexes displayed greater intrinsic kinase activity and affinity for SAMS peptide, α2-complexes were more sensitive to activation by AMP (Figure 4). The α1-complexes required ∼7-fold higher concentration of AMP to achieve comparable allosteric activation as the corresponding α2-complexes (Table 2). A similar AMP dependence of α1- and α2-complexes purified from rat liver was reported earlier [33].

A769662 has been studied extensively as a synthetic, direct AMPK activator. It contains a thienopyridone scaffold with a phenylphenol substituent. Based on a number of studies, it is fairly established that A769662 selectively binds and activates β1-containing AMPK complexes [17,34]. Our biochemical and biophysical studies, carried out against a broad panel of AMPK isoforms, confirm these previous findings. SPR binding studies revealed A769662 binds to α1β1γ1 with a *K*_D_ of 30 nM whereas we could not detect any binding to α2β2γ1 (Figure 5). Similar results were obtained against other β2-containing isoforms, α1β2γ1 and α2β2γ3 (results not shown). Previous mutagenesis, biochemical and structural studies have revealed that A769662 or its chloro-analogue binds at an allosteric interface between α- and β-subunits for functional activation [24,25]. Moreover, the CBM of β2-subunit, which is intrinsically more dynamic compared with β1 CBM, docks on to the kinase domain in a slightly altered pose [23,35]. As a result, A769662 has relatively weaker binding affinity to the α/β allosteric site in β2-containing AMPK complexes and is incapable of functional activation.

Next, we investigated the phosphatase sensitivity and ligand induced protection of pThr^172/174^ from phosphatase (PP2Aα). As expected, the kinase domains (α1-KD and α2-KD) were the most sensitive to phosphatase treatment suggesting that pThr^172/174^ can readily be dephosphorylated in the absence of β- and γ-subunits (Table 1). Among the heterotrimeric complexes, α1-complexes were ∼25-fold more protected from phosphatase than α2-complexes irrespective of the β- and γ-subunits configuration (Figure 6). Among the α1-complexes, however, the α1β2γ3 isoform is slightly (∼3-fold) more sensitive to phosphatase, suggesting that there may be subtle differences in the overall interactions between α1- and γ3-subunits compared with α2- and γ3-subunits. When the protection against phosphatase was studied in the presence of allosteric activators, AMP protected α2-complexes ∼4-fold more than α1-complexes which is consistent with its allosteric activation (Figure 7, Table 3). Also, in agreement with the activation studies, A769662 offered greater protection of β1-containing complexes than β2. While comparing the β1-complexes (α1β1γ1 and α2β1γ1), A769662 significantly enhanced (10- and 3-fold respectively) the protection of pThr^172^ compared with AMP (Table 3). Based on our activation, protection and SPR binding studies it is evident that the allosteric binding interface between α-/β2-subunits is substantially different from the α/β1 interface, resulting in weaker binding and activation by A769662.

Understanding the precise allosteric modulation and activation propensity of AMPK isoforms by small molecule ligands is essential to develop isoform-selective activators of AMPK. In the present study we compared the intrinsic kinase activity, substrate binding kinetics, allosteric protection, modulation and activation of six AMPK isoforms by AMP and A769662. Our studies showed that the intrinsic activity and protection against phosphatase was significantly greater for α1-isoforms than α2-isoforms. However, the α2-isoforms displayed higher AMP-mediated activation efficacy than α1-isoforms. The dose-dependent activation and protection studies along with SPR data conclusively show that A769662 is a weak activator of β2-containing isoforms because of reduced binding affinity at the α/β2 allosteric binding interface. We also noticed that the communication between α- and γ-subunits was reciprocal. Binding of AMP at the γ-subunit protects pThr^172/174^ from phosphatase; at the same time, binding of a ligand at the α-kinase subunit affects one of the AMP-binding sites in the γ-subunit. These studies provide further insights into strategies for the design of specific tissue targeted AMPK activators.

## Abbreviations

α1-KD: α1-kinase domain
AID: auto inhibitory domain
AMPK: AMP-activated protein kinase
CaMKKβ: calmodulin-dependent protein kinase β
CBM: carbohydrate binding module
nt: nucleotide
pAMPK: phosphorylated AMPK
PP2Aα: protein phosphatase 2Aα
SPR: surface plasmon resonance
stau: staurosporine
TCEP: tris-2-carboxyethyl phosphine
